# Microbeam Characterization of Corning Archeological Reference Glasses: New Additions to the Smithsonian Microbeam Standard Collection

**DOI:** 10.6028/jres.107.058

**Published:** 2002-12-01

**Authors:** Edward P. Vicenzi, Stephen Eggins, Amelia Logan, Richard Wysoczanski

**Affiliations:** Smithsonian Institution, National Museum of Natural History, Department of Mineral Sciences, Washington, DC 20560-0119; Research School of Earth Sciences, Australian National University, 0200, Canberra ACT, Australia; Smithsonian Institution, National Museum of Natural History, Department of Mineral Sciences, Washington, DC 20560-0119

**Keywords:** chemical heterogeneity, electron probe microanalysis, glass, laser ablation ICP-MS, microbeam, microscale characterization, secondary ion mass spectrometry

## Abstract

An initial study of the minor element, trace element, and impurities in Corning archeological references glasses have been performed using three microbeam techniques: electron probe microanalysis (EPMA), laser ablation ICP-mass spectrometry (LA ICP-MS), and secondary ion mass spectrometry (SIMS). The EPMA results suggest a significant level of heterogeneity for a number of metals. Conversely, higher precision and a larger sampling volume analysis by LA ICP-MS indicates a high degree of chemical uniformity within all glasses, typically <2 % relative (1 σ). SIMS data reveal that small but measurable quantities of volatile impurities are present in the glasses, including H at roughly the 0.0001 mass fraction level. These glasses show promise for use as secondary standards for minor and trace element analyses of insulating materials such as synthetic ceramics, minerals, and silicate glasses.

## 1. Introduction

### 1.1 Corning Archeological Reference Glasses: Background

While it is uncertain precisely when glass making technology first emerged, glass objects are known from Egypt that are approximately 4500 years old. In the early 1960s there was a growing interest among glass scientists to determining the extent of chemical variation in artifacts from cultures that differed both in time and geography. The ability to compare chemical analyses from laboratories across the globe required a set of high quality reference glasses [1]. This need was articulated during the VIth International Congress on Glass in 1963, and compositions representing the major archeological glass types were proposed [2]. Four glasses were subsequently synthesized at the Corning Glass Works and interlaboratory comparisons of their chemistry were coordinated by Dr. Robert Brill of the Corning Museum of Glass [2–4].

### 1.2 Compositions and Synthesis

The chemistry of the glasses was chosen to mimic four ancient glass varieties and is summarized by Brill [4]. Each glass was doped with between 29 to 31 elements at the major, minor, and trace levels (Table 1). Glasses A and B represent the composition of those objects typical of Egyptian, Mesopotamian, Roman, Byzantine, and Islamic archeological sites and are Na-rich/Ca-bearing silicates. Glass C is rich in Pb and Ba and is similar in composition to glasses found in East Asia. Glass D is a K- and Ca-rich silicate that approximates some younger, so-called medieval glasses, produced from the 17th to 19th centuries.

Precursor materials consisted dominantly of high purity synthetic oxides and carbonates, but also included NaCl, NH_4_H_2_PO_3_, alumina hydrate, zircon (natural ZrSiO_4_), and “African sand” as a source of SiO_2_ [4]. Trace elements were mixed in 2 groups (*#*1: Ti, Sn, B, Ba, Sr, Li, and Rb; *#*2: V, K, Ag, Zr, Ni, Zn, and Bi) and ball-milled for 16 h prior to incorporation into 15 kg batches. This method allowed for constant trace element ratios of metals (within each trace element group) among the four compositions. The precursor mixtures were then melted in platinum crucibles and held at 1450 °C for 3 h to 4 h. Melts were stirred with Pt rods and quenched in deionized water. The glasses were then lightly crushed and re-melted at 1450 °C for another 2 h. The rehomogenized melts were also stirred and finally poured out as 1 cm thick sheets, which were annealed at 450 °C.

Participants of analytical round-robins involving these glasses have employed a number of classical bulk chemical techniques, including: gravimetry, volumetry, colorimetry, polarography, flame photometry, and redox titration [4]. Atomic absorption, x-ray fluorescence, neutron activation, and emission spectroscopies have also been conducted to determine the composition of many of the element abundances in the reference glasses [3].

### 1.3 Microbeam Characterization

Although significant effort was spent during the fabrication process to ensure homogeneity in the glasses, an examination of glass chemistry on the submillimeter to micrometer length scale has not been performed. We used three microbeam techniques, electron probe microanalysis (EPMA), laser ablation inductively coupled-mass spectrometry (LA ICP-MS), and secondary ion mass spectrometry (SIMS) to determine (to first order) the minor and trace element characteristics of the glasses. Once the degree of spatial heterogeneity of the minor and trace elements has been established, these materials can be considered for duel purposes in laboratories using microchemical techniques: 1. for use as a secondary standards, and 2. for use as a primary standard for the analysis of elements where more desirable forms (e.g., simple oxides, phosphates, or other stoichiometric compounds) are not available. All four Corning glasses were recently accessioned into the Smithsonian Microbeam Standard (SMS) collection [5] and hence are available for distribution to research laboratories for follow-up and additional characterization.

## 2. Electron Probe Microanalysis (EPMA)

X-ray microanalysis was performed with a five-wavelength dispersive spectrometer (WDS) instrument to analyze for S, Cl, Co, Cu, Zn, Sr, Sn, Sb, Ba, and Pb in glass. An accelerating voltage of 15 keV was used to measure K-line (S, Cl, Co, Cu,and Zn), L-line (Ba, Sn, Sr, and Sb), and M-line (Pb) radiation using either LiF or PET diffracting crystals. A combination of well-characterized minerals and synthetic materials were used to calibrate for EPMA, including SMS scapolite (S and Cl), Corning W glass [6] (Co), SMS gahnite (Zn), SMS cassiterite (Sn), SMS strontianite and Corning X glass (Sr). In the absence of a suitable primary standard, some elements were “self calibrated” using the Corning archeological glasses themselves, for example, Glass B (Cu), Glass A (Sb), and Glass C (Ba and Pb). Corrections were made for the following first order peak interferences: CuK_β_ on ZnK_α_ , SiK_β_ on SrL_α_ , KK_β_ on SbL_α_, and raw data were reduced using a ZAF matrix correction routine [7].

In order to establish the appropriate electron beam conditions to conduct the analyses, a series of measurements were made to determine the time dependence of Na, K, and SiK_α_ x-ray intensities at a variety of current densities. Using a beam current of 20 nA and a beam diameter of 10 µm, both the Na and K intensities dropped in Glass A as a function of time while the Si intensities rose in a complementary manner. This behavior suggests that heating resulting from energy lost by the electron beam caused interdiffusion of alkalis and silicon about the interaction volume. Conversely, a 30 nA beam spread over a 30 µm diameter produced stable intensities for all measured x rays. Because each measurement for minor and trace elements was moderately long (120 s on peak and 60 s off peak position) beam conditions of 30 nA and 40 µm were chosen for all analyses.

**Fig. 1:**
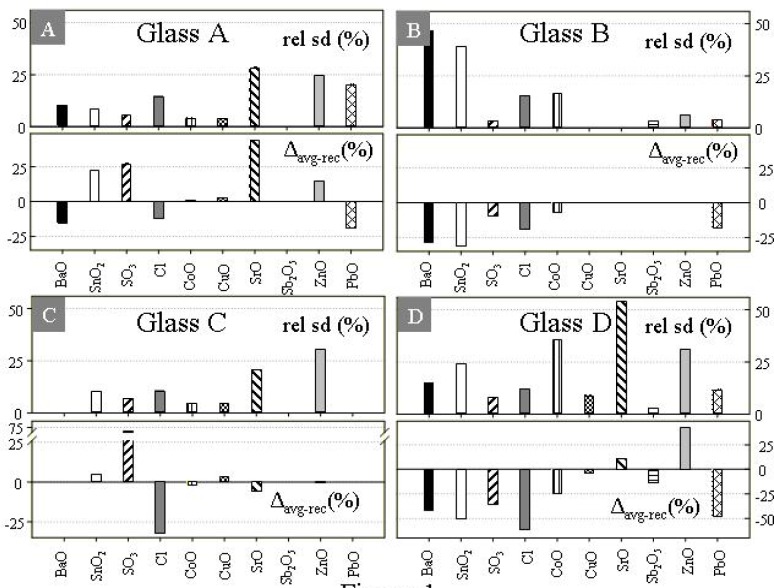
Upper plots represent the homogeneity of glasses A, B, C, and D expressed as 1 σ relative (%) of EPMA analyses. Lower plots show the relative difference between the recommended bulk and EPMA values. Measurement times of 120 s on peak/60 s off peak were used with a 40 µm electron beam composed of 30 nA of current.

On the basis of EPMA data alone the Corning glasses appear to be heterogeneous with respect to a number of metals (Table 2). Plots of the relative standard deviation of measurements (n typically 100) indicate that SrO and ZnO appear to be the most poorly homogenized across all compositions (Fig. 1a–d: upper plots). BaO and SnO_2_ are particularly heterogeneous in glass B, and less so in glasses D and A. Conversely, the concentrations of Sb_2_O_5_, CuO, and SO_3_ cluster more tightly in all glasses. Figure 1(a–d: lower plots) also shows that the agreement of the EPMA data with published bulk values is highly variable. In general, the x-ray microanalysis values are typically lower than the published recommended concentrations for most metal oxides. Particularly poor agreement for SO_3_ concentrations may be attributed to the fact that both the Cl and SO_3_ are not known to a high degree of certainty, and were originally estimated by assuming incomplete retention of the their precursor chloride and sulfate during synthesis [4].

## 3. Laser Ablation Inductively Coupled-Mass Spectrometry (LA ICP-MS)

The ArF (193 nm) EXCIMER laser sampling system and ICP-MS instrumentation employed for analysis have been described in detail elsewhere [8, 9]. A broad (25 mm×8 mm) UV laser beam is used to illuminate an aperture, the image of which is demagnified 20 times onto the sample surface using a 150 mm focal length, UV-grade-silica doublet lens. Application of a laser fluence of 15 J/cm^2^ results in uniform ablation and removal of ≈100 nm from the target material per laser pulse. Ablation is conducted in a He atmosphere in order to minimize sample recondensation during ablation about the target site and thereby maximize sample transport to the ICP [9]. The He flow (300 cm^3^/min) containing the ablation products was subsequently combined with the main Ar carrier flow (≈1000 cm^3^/min) prior to delivery via a signal smoothing device that damps the intrinsic laser pulsations in the signal, into the ICP.

The ICPMS instrument was operated in two modes of analysis. The first was optimized for maximum sensitivity on ^43^Ca, ^88^Sr, and ^238^U by rastering (scanning) a 65 µm circular spot at a laser pulse repetition rate of 5 pulses/s across the NIST SRM 612 glass [10] while maintaining ThO^+^/Th^+^ ratios <0.5 %. This mode is designed to measure very low concentrations of trace elements and impurities. For these “impurity” measurements on the Corning glasses, a 65 µm stationary spot was used and ions were collected for 60 s. Sensitivities for analyte elements vary subject to the ionization efficiency, isotope abundance and position in the mass spectrum (with sensitivity generally increasing with mass). Eleven trace and minor elements (^7^Li, ^11^B, ^51^V, ^53^Cr, ^60^Ni, ^85^Rb, ^88^Sr, ^90^Zr, ^138^Ba, ^204^Pb, ^209^Bi) and 16 impurities were measured in this mode of operation (^9^Be, ^45^Sc, ^71^Ga, ^89^Y, ^93^Nb, ^95^Mo, ^133^Cs, ^140^Ce, ^151^Eu, ^175^Lu, ^178^Hf, ^181^Ta, ^195^Pt, ^205^Tl, ^232^Th, and ^238^U). The second mode of operation involves rastering the sample with respect to the laser spot using a motorized stage driven at 1 mm/min. In this instrument setup, ablated material is sampled continuously across the entire transect length of a polished glass shard (e.g., 3 mm to 4 mm). For the raster analyses in this study, a 23 µm diameter laser ablation spot and 20 Hz laser pulse rate was employed. Twelve minor and trace elements were measured (^31^P, ^47^Ti, ^55^Mn, ^57^Fe, ^65^Cu, ^68^Zn, ^88^Sr, ^118^Sn, ^121^Sb, ^137^Ba, and ^208^Pb). Major elements were also collected but are not reported here. Most analyte isotope sensitivities range between ≈150 and 1000 counts ×s^−1^ mass fraction ×10^6^ in the laser rastering setup and between 5000 and 20 000 counts ×s^−1^ × mass fraction ×10^6^ in the point analyses.

All standards and unknowns were analysed using a point analysis procedure. ^43^Ca was employed as the internal standard based on recommended bulk CaO concentrations [11]. A drift correction was applied to the unknowns between the bracketing NIST SRM 612 calibrations, assuming a linear variation in measured signal intensity ratios with the analysis sequence. The analysis protocol involved external calibration of the instrument using the NIST SRM 610 glass (raster analyses) and NIST SRM 612 (point analyses) and subtraction of “gas” (laser off) background count rates from all measured signal intensities. All reference materials and background measurements were measured for a period of 90 s, operating the ICP-MS in simultaneous pulse counting/peak hopping mode, and acquiring data on a single point per isotope using 20 ms dwell times for most internal standard and analyte isotopes.

**Fig. 2:**
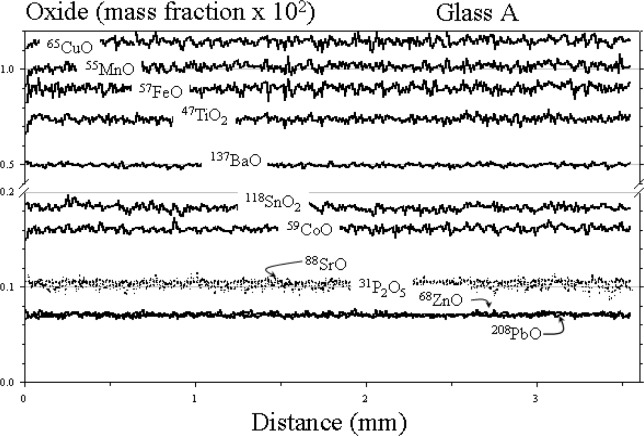
LA ICP-MS minor and trace element profiles by raster mode, or continuous grid sampling of glass A. The sample was translated at 1 mm/min under a 23 µm laser spot operated at 20 Hz laser pulse rate.

**Fig. 3:**
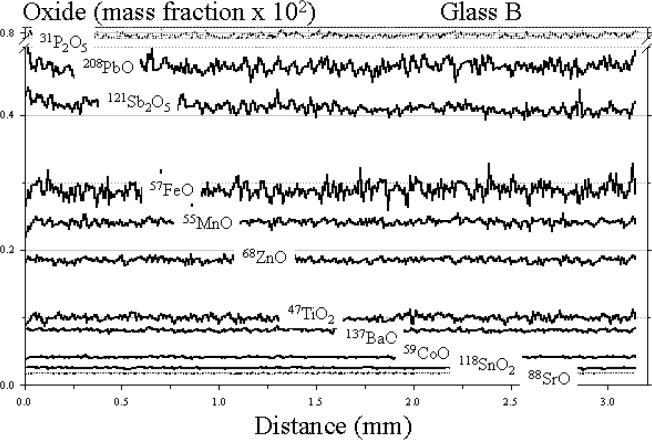
LA ICP-MS minor and trace element profiles by raster mode, or continuous grid sampling of glass B. The sample was translated at 1 mm/min under a 23 µm laser spot operated at 20 Hz laser pulse rate.

**Fig. 4:**
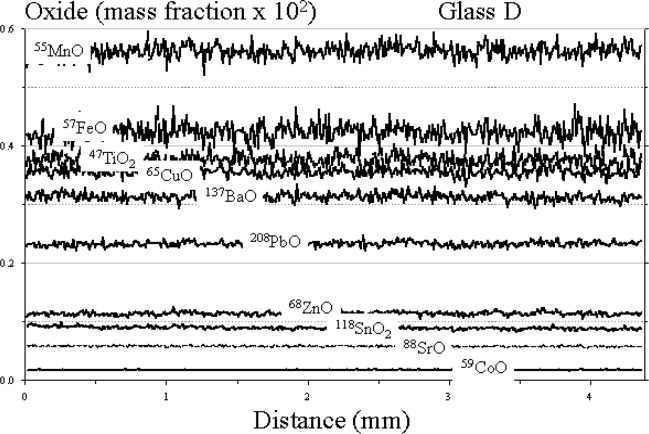
LA ICP-MS minor and trace element profiles by raster mode, or continuous grid sampling of glass D. The sample was translated at 1mm/min under a 23 µm laser spot operated at 20 Hz laser pulse rate.

The results of the raster analysis for glasses A, B, and D are shown in Figs. 2, 3, and 4, respectively. Because this method of sampling is virtually continuous and covered many millimeters of the polished surface, these data represent the best estimate for determining microchemical heterogeneity in the glasses. The profiles of all oxides show remarkable degree of homogeneity for minor and trace components. Relative standard deviations of analyses are typically less than 2 % (1 σ) and often less than 1 %. Agreement between the raster LA ICP-MS values and published bulk values is highly variable but typically within 5 % to 20 %.

**Fig. 5:**
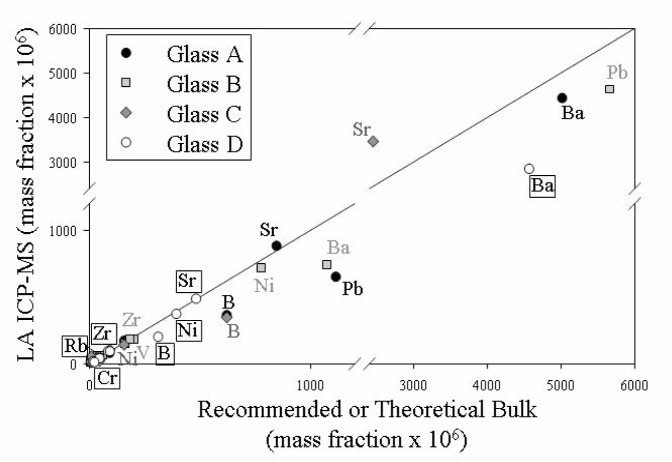
LA ICP-MS results versus either the recommended, or theoretical bulk values for minor and trace elements obtained by spot mode analysis. A 65 µm stationary laser spot was used to collect ions for 60 s/measurement.

Only three spot mode analyses were collected from each glass so information regarding heterogeneity is strictly limited. In broad terms, the trace elements measured in this style of operation showed better agreement with the recommended or theoretical published values for the bulk glasses than were minor elements (Fig. 5). Nearly all impurities are present at the level of less than 10^−6^ mass fraction. (Table 3). Pt which was used in the synthesis process is found in single mass fraction ×10^6^ abundances, though it can be traced to the stirring elements used during glass synthesis.

## 4. Secondary Ion Mass Spectrometry (SIMS)

A magnetic sector secondary ion mass spectrometer using a 25 µm diameter Cs^+^ primary ion beam was used to measure ^32^S and ^35^Cl, as well as impurity levels of ^1^H, ^12^C, and ^19^F. Secondary ions were extracted in the negative polarity, measured at a mass resolution of ≈2400 m/Δm (full width at half maximum), and detected with an electron multiplier. The instrument was calibrated using volcanic glasses standards and procedures described by Hauri et al. [12].

**Fig. 6:**
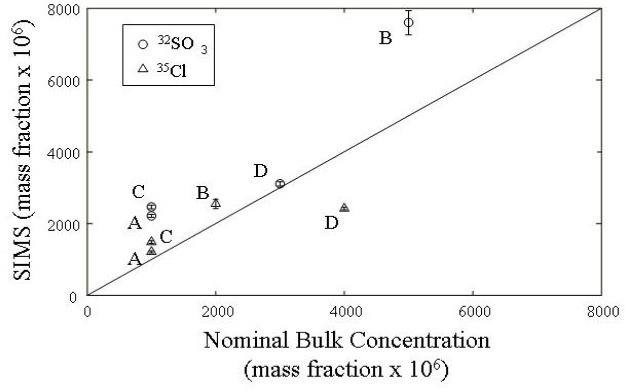
SIMS results versus the nominal bulk values for SO_3_ and Cl. A 25 µm Cs^+^ primary ion beam was used to sputter the sample surface. Negative secondary ions were collected using a moderate mass resolution and an electron multiplier ion detector.

Six analyses per glass were collected. The relationship between the SIMS results and nominal bulk concentrations of S and Cl are shown in Fig. 6. As discussed above, the lack of a better correlation likely stems from uncertainty in the degree of volatile loss during glass synthesis. Volatile impurities are listed in Table 4. A small but measurable amount of hydrogen is present in all glasses. Despite high temperature processing H (expressed as H_2_O) was stable in the silicate melts at the 10^−4^ mass fraction level at room pressure. A double polished thin section of each glass was made, and transmission Fourier transform infrared spectra were collected that confirmed the presence of hydroxyl in the glasses.

## 5. Summary

While EPMA offers the highest spatial resolution of the three methods employed, the measurement times become impractically long when attempting to measure large groups of elements present at the 0.0001 mass fraction to 0.001 mass fraction level. This fact may account for the discrepancy between the fairly large degree of chemical heterogeneity in the glasses suggested by EPMA, relative to the high degree of compositional uniformity indicated by raster mode LA ICP-MS. Because LA ICP-MS is a high precision method, coupled with the significantly larger volume of material sampled in the raster mode, we can state with certainty that these glasses show promise for use as secondary standards of minor and trace elements. Because these materials are now part of the Smithsonian Microbeam Standard collection, it is hoped that research laboratories will request material to perform follow-up microbeam characterization of the archeological reference glasses.

## Figures and Tables

**Table 1:** Major, minor, and trace element compositions of glasses (Brill, 1999) mass fraction ×10^2^

Corning USNM #	A 117218.004	B 117218.001	C 117218.002	D 117218.003
SiO_2_	66.56^a^	61.55^a^	34.87^a^	55.24
Al_2_O_3_	1.00	4.36	0.87	5.30
Fe_2_O_3_	1.09	0.34	0.34	0.52
MgO	2.66	1.03	2.76	3.94
CaO	5.03	8.56	5.07	14.8
Na_2_O	14.3	17.0	1.07	1.20
K_2_O	2.87	1.00	2.84	11.3
MnO	1.00	0.25	0.82^a^	0.55
P_2_O_5_	0.13	0.82	0.14	3.93
TiO_2_	0.79	0.089	0.79	0.38
Sb_2_O_5_	1.75	0.46	0.03	0.97
CuO	1.17	2.66	1.13	0.38
PbO	0.12	0.61	36.7	0.48
CoO	0.17	0.046	0.18	0.023
BaO	0.56	0.12	11.4	0.51
SnO_2_	0.19	0.04	0.19	0.10
SrO	0.10	0.019	0.29	0.057
ZnO	0.044	0.19	0.052	0.10

Nominal compositions^b^				

B_2_O_3_	0.20	0.02	0.20	0.10
Li_2_O	0.01	0.001	0.01	0.005
Cl	0.10	0.2	0.10	0.4
SO_3_	0.10	0.5	0.10	0.3
Rb_2_O	0.01	0.001	0.01	0.005
V_2_O_5_	0.006	0.03	0.006	0.015
Cr_2_O_3_	0.001	0.005	0.001	0.0025
NiO	0.02	0.10	0.02	0.05
ZrO_2_	0.005	0.025	0.005	0.0125
Ag_2_O	0.002	0.01	0.002	0.005
Bi_2_O_3_	0.001	0.005	0.001	0.0025

Total	99.94	99.87	99.95	100.59

a From Brill (unpublished data).

b Calculated from precursor mass fractions.

**Table 2:** Minor (and trace) element compositions of glasses by EPMA in mass fraction ×10^2^

	*n*	A Avg	Sd (1 σ)	Rel sd (%)	*n*	B Avg	Sd (1 σ)	Rel sd (%)	*n*	C Avg	Sd (1 σ)	Rel sd (%)	*n*	D Avg	Sd (1 σ)	Rel sd (%)
BaO	105	0.47	0.05	10.1	102	0.09	0.04	46.7					152	0.30	0.04	15.0
SnO_2_	105	0.23	0.02	8.8	102	0.03	0.01	38.8	101	0.20	0.02	10.0	152	0.05	0.01	24.1
SO_3_	75	0.13	0.01	5.5	40	0.45	0.02	3.4	50	0.17	0.01	6.7	40	0.19	0.02	8.1
Cl	105	0.09	0.01	14.5	102	0.16	0.02	15.3	101	0.07	0.01	10.6	152	0.16	0.02	11.9
CoO	105	0.17	0.01	4.2	102	0.04	0.01	16.3	101	0.18	0.01	4.6	152	0.02	0.01	35.5
CuO	105	1.20	0.05	3.8					101	1.17	0.05	4.3	152	0.36	0.03	8.8
SrO	75	0.14	0.04	28.3	72	bd			71	0.27	0.06	20.5	80	0.06	0.03	54.1
Sb_2_O_5_					72	0.46	0.01	3.1	71	bd			80	0.84	0.02	3.0
ZnO	10	0.05	0.01	24.7	10	0.19	0.01	6.0	10	0.05	0.02	30.3	80	0.14	0.04	30.8
PbO	75	0.10	0.02	20.3	40	0.50	0.02	3.6					40	0.25	0.03	11.7

**Table 3:** Impurities in glass by spot mode LA ICP-MS in mass fraction ×10^6^

Glass *n*	A 3		B 3		C 3		D 3	
	Avg	Sd	Avg	Sd	Avg	Sd	Avg	Sd
^9^Be	0.06	0.01	0.07	0.003	0.02	0.005	0.03	0.02
^45^Sc	0.566	0.009	0.549	0.020	0.311	0.028	0.496	0.004
^71^Ga	0.595	0.010	2.43	0.02	0.405	0.003	2.38	0.04
^89^Y	0.365	0.008	0.474	0.007	4.284	0.084	0.370	0.002
^93^Nb	0.598	0.003	0.179	0.003	0.744	0.004	0.559	0.008
^95^Mo	3.23	0.01	1.66	0.03	3.30	0.40	3.32	0.11
^133^Cs	0.255	0.005	0.061	0.002	0.368	0.017	0.140	0.001
^140^Ce	0.236	0.002	0.164	0.003	0.046	0.001	0.256	0.002
^151^Eu	0.012	0.001	0.004	0.001	0.126	0.004	0.007	0.001
^175^Lu	0.005	0.001	0.019	0.001	0.024	0.001	0.011	0.001
^178^Hf	0.949	0.007	4.152	0.018	1.677	0.011	2.115	0.033
^181^Ta	0.124	0.003	0.089	0.003	0.120	0.002	0.231	0.004
^195^Pt	4.21	0.68	1.33	0.01	8.56	2.89	0.80	0.0
^205^Tl	0.06	0.01	0.20	0.01	18.53	0.49	0.10	0.00
^232^Th	0.288	0.001	0.805	0.013	0.204	0.003	0.648	0.005
^238^U	0.1823	0.0025	0.2258	0.0046	0.0786	0.0013	0.1603	0.0014

**Table 4:** Impurities in glass by SIMS in mass fraction ×10^6^

Glass	A	B	C	D
^1^H_2_O	180	320	310	290
1 σ	20	20	20	20
^12^CO_2_	bd	bd	bd	bd
^19^F	34.0	69.6	33.1	45.9
1 σ	0.5	2.5	0.5	0.9
